# Suicidal and aggressive behavior among populations within institutional quarantine and isolation centers of COVID-19 in eastern Ethiopia: A cross-sectional study

**DOI:** 10.1371/journal.pone.0287632

**Published:** 2023-06-27

**Authors:** Tadesse Misgana, Dejene Tesfaye, Mandaras Tariku, Tilahun Ali, Daniel Alemu, Yadeta Dessie

**Affiliations:** 1 Department of Psychiatry, College of Health and Medical Sciences, Haramaya University, Harar, Ethiopia; 2 School of Public Health, College of Health and Medical Sciences, Haramaya University, Harar, Ethiopia; University of Nairobi, KENYA

## Abstract

**Introduction:**

The coronavirus disease is still not under the control globally and has caused various mental health problems such as depression, anxiety, suicide, and aggressive behavior in different populations. The pandemic-related issues which are applied to control the pandemic such as protection measures against COVID-19, social distancing, isolation, and quarantine can also trigger mental health problems.

**Objective:**

This study aimed to assess suicidal behavior and aggression, and its correlates during COVID-19 among populations within institutional quarantine and isolation centers in Ethiopia.

**Method:**

A cross-sectional study was conducted among a sample of 392 participants. The convenience sampling method was used to select the study participants. Suicide Behavioral Questionnaire-Revised (SBQ-R) and the Modified Overt Aggression Scale (MOAS)were applied to determine the suicide and aggressive behavior of study participants respectively. Epi-data 3.1 and SPSS 20.0were used to enter and analyze the data respectively. Logistic and linear regressions were fitted to explore correlates associated with suicidal behavior and aggression respectively.

**Results:**

The prevalence of suicidal behavior was 8.7% (95% CI: 6.1, 11.5) whereas the mean total score of behavioral aggression was 2.45±5.90 (95% CI: 1.84, 3.08). Being female (AOR = 2.63, 95% CI: 1.09, 6.32), having common mental disorders (AOR = 6.08, 95% CI: 2.32, 15.93), manifesting the symptoms of COVID-19 (AOR = 2.17, 95% CI: 1.48, 2.86), and poor social support (AOR = 7.30, 95% CI: 1.44, 37.10) were significantly associated with suicidal behavior, whereas male gender (β coefficient = 3.0, 95% CI: 1.35, 4.70), low level of knowledge about COVID-19 (β coefficient = 1.87, 95% CI: 1.09, 3.41), and substance use (β coefficient = 1.7, 95% CI: 1.23, 6.47) were positively associated with mean overt aggression score.

**Conclusion:**

The present study revealed that suicidal and aggressive behaviors were prevalent with significant correlates. Therefore, it is important and required to provide focused mental health and psycho-social services for the selected and high-risk populations such as those in quarantine and isolation centers for being suspected.

## Introduction

The WHO declared COVID-19 as a pandemic a year ago [1] and has become a global concern [2]. Since the beginning, various countries have been struggling to mitigate the spread of the pandemic through different protection measures such as quarantine and isolation, massive lockdowns, and vaccines. Currently, there is no exact prediction for how long the pandemic will continue, how many people will be infected, or, consequently, how long people’s lives will be troubled [3, 4]. As observed in preceding epidemics and pandemics, in addition to burdening the health system, the threat and insecurity surrounding public protection, which is difficult to predict, can have negative consequences on individuals’ mental health like suicide and aggressive behavior [5, 6].

In addition to this, the protection measures taken against COVID-19 are applied to control the pandemic like mass quarantine and isolation, and consequently, the social and economic fallout can precipitate mental health problems [1, 7]. With such long-lasting suffering and severe condition, mental health problems can result in suicidal-related thoughts and behaviors [8, 9]. The pandemic could adversely affect other known precipitants of suicide [10]. For instance, intimate partner violence and drinking alcohol might rise during the period of lockdown [11].

A strong association has been seen between the increase in suicide and traumatic events such as the viral pandemic. In one systematic review study, suicidal-related behavior increased at the time of COVID-19 pandemic as compared to the pre-COVID-19 pandemic period [12]. Another recent study conducted in Iran during the COVID-19 Pandemic shows that the magnitude of suicidal thoughts was increased to 20.8% [13]. In earlier pandemics, similar conditions were reported [5]. The suicide-related consequences of the pandemic might vary depending on countries’ public health control measures, socio-cultural and demographic structures, availability of digital alternatives to face-to-face consultation, and existing supports. The effects might be worse in resource-poor settings like Ethiopia where economic adversity is compounded by inadequate welfare support.

If the extended restriction of personal movement and disruption in daily life contribute to hindering individual goals, it would logically follow that aggression would increase during lockdowns [14]. In support of this, intimate partner aggression perpetration is associated with pandemic-related negative outcomes, such as social isolation [15] and economic stressors [16]. Taken together, this literature provides indirect support for the view that people who experience heightened COVID-19 stress are at greater risk for intimate partner aggression perpetration. However, no studies have yet directly addressed suicidal behaviors and levels of measured aggression as a function of lockdown status during the COVID-19 pandemic in Ethiopia. Therefore, this study aimed to assess suicidal behavior, aggression, and its correlates during the COVID-19 pandemic among populations within institutional quarantine and isolation centers.

## Materials and methods

### Study setting, design, and period

A multi-center cross-sectional study was done in the Harari regional state and eastern Hararghe zone of Oromia regional state, eastern Ethiopia and the participants were recruited from November 15 to December 31, 2020. Ethiopia has established a COVID-19 task force as part of the response to the pandemic. In addition to selected health facilities for the treatment of patients with symptomatic COVID-19, there were isolation centers that catered to the infected but asymptomatic individuals. Furthermore, there were designated quarantine centers for suspected cases and those awaiting test results.

### Population and eligibility

Suspected cases of COVID-19 aged 18 and above in quarantine and isolation centres were considered as the study population, and those who were unable to participate due to serious underlying health conditions or severe symptoms of COVID-19 were excluded.

### Sample size determination

The sample size was estimated using two approaches based on the objectives of the study. Accordingly, for each specific objective, the sample size is calculated separately and the larger sample size was taken to be used for this study. For the first objective, to estimate the prevalence of suicidal behavior, a single population proportion formula was used with the following assumptions: prevalence of suicidal behavior 50% since there is no study conducted during the COVID-19 pandemic, a 5% margin of error, and a 5% of non-response rate. The sample size was 422. The sample size for the second specific objective is determined by considering factors that are significantly associated with the suicidal behavior (being female), the two-sided confidence level of 95%, the margin of error of 5%, power of 80%, and the ratio of exposed to unexposed 1:1 using STAT CALC of Epi Info Version 7. Finally, the required sample size for this particular study is decided by taking the maximum from the calculated sample size, which is422. In this study, the latter sample size was considered to increase the power of the study.

### Sampling technique and procedure

From two isolation centers and 8 quarantine centers in Harari regional state, randomly we selected one isolation center and three quarantine centers. Again, the East Hararghe zone of Oromia regional state had one isolation center and 12 quarantine centers, and we randomly selected 4 of them. Finally, the participants were conveniently selected from each center proportionally based on the number of suspected cases they occupied. The data were collected in face-to-face by considering all the essential precautions recommended by WHO to control the transmission of COVID-19.

### Data collection instruments

The semi-structured questionnaire which contains 8 sections was used. The questionnaires for assessing the socio-demographic and clinical factors were adopted by reviewing the literature. The Self-Reporting Questionnaire (SRQ-20) was used to determine the presence of common mental disorders (CMD).The SRQ-20 was validated in Ethiopia at a general population [17]. Alcohol, Smoking, and Substance Involvement Screening Test (ASSIST) [18] and Oslo 3-item Social Support Scale (OSSS-3) [19] were administered to assess the substance use and social support of the participants respectively.

Suicide Behavioral Questionnaire-Revised (SBQ-R) was used to screen the suicidal behaviors of study participants. Each item assesses a different dimension of suicidality (or risk of suicide) and the higher the obtained score reflects the higher risk for subsequent suicidal behavior. SBQ-R is made up of 4 items scale ranging from 3 to 18, with a cutoff score of ≥7 showing the presence of suicidal behavior [20]. In this study, the reliability of SBQ-R was checked and it was a kappa value of 0.91.

The Modified Overt Aggression Scale (MOAS) was used to screen the level of aggression among suspected cases at quarantine and isolation centers. MOAS has a total of 16 items with four categories that assesses aggression, namely verbal aggression, aggression against property, auto-aggression, and physical aggression. The severity of aggressive behavior ranges from 0 (no aggression) to 4 points (maximum violence) for each category or it means each domain is multiplied by the number of the category ranging from 0 (no aggression) to 40 (maximum aggression) [21].The MOAS has been tested at various centers in the developed world and found to be a valid indicator of the type and severity of aggression with good inter-rater reliability. The MOAS has excellent psychometric properties; the correlation coefficient between raters was 0.9. Furthermore, it is validated in Nigeria [22].

### Data quality control

The questionnaire was prepared first in the English language and translated to local languages (Afan Oromo, and Amharic languages) and back to the English language by professional translators to maintain the consistency of the data collection tool. Standardized tools specific to the research objectives were adopted and intensive training was given to the data collectors and supervisors. A pre-test was done on 5% of the sample size in Dire Dawa city administration in order to evaluate the acceptability and applicability of the procedures and tools. Data collectors were closely supervised during the data collection and two data clerks were doing the data entry to minimize errors during data entry.

### Data processing and analysis

The data were entered and analyzed using Epidata3.1 and SPSS 20.0 respectively. Absolute frequencies and percentages were calculated for categorical variables, and mean and standard Deviation were calculated for continuous variables. Whenever the data were not normally distributed, median and IQR were calculated. Bi-variable and multivariable logistic regression was carried out to determine associations of suicidal behavior and independent variables. Fourteen variables were included during bivariate analysis and seven variables with P-value ≤ 0.25 was taken into consideration in the multivariable model to control for all possible confounders. Linear regression was performed for the total score of the behavioral aggression to investigate the association with different explanatory variables. In simple linear regression, twelve variables were included and seven were included in the final multiple regression model based on their significance level. The adjusted R2, the unstandardized B coefficient with its 95% CI, and the p-value were calculated. For both tests, the significance level was set at a p-value of 0.05.

### Ethics consideration

The study was conducted following the principles of the Declaration of Helsinki. Ethical clearance was obtained from the Institutional Health Research Ethics Review Committee (IHRERC) of the College of Health and Medical Sciences, Haramaya University with the approval number of IHRERC/243/2020. Written informed consent was obtained from the participants and those identified as high risk of suicidal and aggressive behavior were referred to a psychiatry specialty clinic for advance assessment and management.

## Results

### Socio-demographic descriptions

Of 423 participants, the response rate was 92.7% (392 participants). About 205 (52.3%) were males, with a median age of 30.5(IQR = 10.6) years. About 204 (52%) of the participants were married and 39 (9.9%) indicated they could not read and write (Table 1).

**Table 1:** Distribution of socio-demographic characteristics of populations within institutional quarantine and isolation centers of COVID-19 in eastern Ethiopia, 2020 (N = 392).

Variables	Frequency	Percentage (%)
**Sex**		
Male	205	52.3
Female	187	47.7
**Age (years)**		
<30	244	62.2
30–39	81	20.7
40–49	47	12.0
≥50	20	5.1
**Marital status**		
Never married	179	45.7
Married	204	52.0
Others*	9	2.3
**Educational status**		
Can’t write and read	39	9.9
Only able to write and read	74	18.9
Primary school	63	16.1
Secondary and preparatory school	101	25.8
College and above	115	29.3

Others* = divorced, widowed, separated

### Clinical descriptions

About 2.0% had a previous history of known mental illness and 18.9% didn’t put into practice any COVID-19 control measures. In another way, 227 (57.9%) of the participants know how to protect themselves from the disease while 193 (49.2%) know how it is transmitted. About 101 (25.8%) participants had inadequate accessibility to personal protective equipment and 205 (52.3%) heard about COVID-19 twice daily. One-third (27%) of the participants manifested the typical symptoms of COVID-19 (Table 2).

**Table 2:** Distribution of clinical characteristics of populations within institutional quarantine and isolation centers of COVID-19 in eastern Ethiopia, 2020 (N = 392).

Variables	Frequency	Percentage (%)
**History of mental illness**		
Yes	8	2.0
No	384	98.0
**History of chronic medical illness**		
Yes	25	6.4
No	367	93.6
**Practicing COVID-19 control measures**		
Yes	74	18.9
No	318	81.1
**Accessibility of PPE**		
Yes	291	74.2
No	101	25.8
**COVID-19 information**		
None	71	18.1
2 times daily	205	52.3
3 and more times daily	116	29.6
**Symptoms of COVID-19**		
Yes	106	27.0
No	286	73.0
**Perceived knowledge about COVID-19**		
Poor	130	33.2
Good	262	66.8

### Psycho-social and substance use descriptions

About 16.5%, 86.5%, and 16.5% of the respondents were reporting that they used tobacco, khat, and alcohol in the previous three months respectively, whereas 165 (42.1%) had received good social support. About 13.5% of the participants had common mental disorders as screened using SRQ-20 (Table 3).

**Table 3:** Distribution of psycho-social and substance-related characteristics of populations within institutional quarantine and isolation centers of COVID-19 in eastern Ethiopia, 2020 (N = 392).

Variables	Frequency	Percentage (%)
**Social support**		
Poor	57	14.5
Moderate	170	43.4
Strong	165	42.1
**Common mental disorders**		
Yes	53	13.5
No	339	86.5
**Lifetime Alcohol use**		
Yes	79	20.2
No	313	79.8
**Lifetime Tobacco use**		
Yes	71	18.0
No	321	82.0
**Lifetime khat use**		
Yes	366	93.4
No	26	6.6
**Current Alcohol use**		
Yes	65	16.5
No	327	83.5
**Current Tobacco use**		
Yes	65	16.5
No	327	83.5
**Current khat use**		
Yes	339	86.5
No	53	13.5

### Suicidal behavior

The reported prevalence of suicidal behavior among suspected cases of COVID-19 was 8.7% (95% CI: 6.1, 11.5) (SBQ-R≥7). The lifetime prevalence of suicidal ideation, plan, and attempt was 21.9%, 4.8%, and 2.3%, respectively. The 12-monthprevalence of suicidal ideation was 8.0% (95% CI: 5.2, 10.7). The mean SBQ-R score was 3.88 with a standard deviation of 2.26.

### Behavioral aggression (The Modified Overt Aggression Scale)

According to the Modified Overt Aggression Scale (MOAS), the mean total score for behavioral aggression was 2.45±5.90 (95% CI: 1.84, 3.08). About 20.4% of the participants scored above the mean of the overt aggression scale. The 4 main categories were described in Table 4.

**Table 4:** Description of the categories of behavioral aggression among populations within institutional quarantine and isolation centers of COVID-19 in eastern Ethiopia, 2020 (N = 392).

Categories	Mean ± Standard deviation	95% CI of mean
**Verbal aggression**	0.23±0.59	0.17, 0.29
**Aggression toward objects**	0.47±1.23	0.35, 0.59
**Auto-aggression**	0.81±2.06	0.62, 1.02
**Physical aggression**	0.93±2.91	0.65, 1.22

### Factors associated with suicidal behavior

Bi-variable logistic regression model revealed that suicidal-related behavior was significantly associated with age, sex, chronic medical illness, symptoms of COVID-19, common mental disorders (CMD), social support, and use of the substance. However during multivariable logistic regression analysis, female gender (AOR = 2.63, 95% CI: 1.09, 6.32), having CMD (AOR = 6.08, 95% CI: 2.32, 15.93), manifesting symptoms of COVID-19 (AOR = 2.17, 95% CI: 1.48, 2.86),and poor social support (AOR = 7.30, 95% CI: 1.44, 37.10) were significant associated with suicide (Table 5).

**Table 5:** Bivariate and multivariate logistic regression analysis of factors associated with suicidal behavior among populations within institutional quarantine and isolation centers of COVID-19 in eastern Ethiopia, 2020 (N = 392).

Study Variables	Suicidal behavior			COR (95% CI)	AOR (95% CI)		P-values
	With suicidal behavior		Without suicidal behavior				
**Sex**							
Male	9 (4.4%)	196 (95.6%)		1	1		
Female	25 (13.4%)	162 (86.6%)		3.36 (1.53, 7.40)	2.63 (1.09, 6.32)		0.03
**Age (in years)**							
<30	16 (6.6%)	228 (93.4%)		1	1		
30–39	8 (9.9%)	73 (90.1%)		1.56 (0.64, 3.79)	1.19 (0.40, 3.53)	0.74	
40–49	6 (12.8%)	41 (87.2%)		2.08 (0.77, 5.64)	1.64 (0.49, 5.41)	0.41	
≥50	4 (20.0%)	16 (80%)		3.56 (1.07, 11.91)	2.49 (0.59, 10.39)	0.21	
**Chronic medical illness**							
No	28 (7.6%)	339 (92.4%)		1	1		
Yes	6 (24.0%)	19 (76.0%)		3.82 (1.41, 10.35)	2.23 (0.57, 8.67)	0.24	
**Symptoms of COVID-19**							
No	18 (6.3%)	268 (93.7%)		1	1		
Yes	16 (15.1%)	90 (84.9%)		2.65 (1.29, 5.41)	2.17 (1.48, 2.86)	0.01	
**Common mental disorders**							
**No**	17 (5.0%)	322 (95.0%)		1	1		
**Yes**	17 (32.1%)	36 (67.9%)		8.94 (4.20, 19.03)	6.08 (2.32, 15.93)	<0.001	
**Substance use**							
No	21 (7.0%)	281 (93.0%)		1	1		
Yes	13 (14.4%)	77 (85.6%)		2.25 (1.08, 4.71)	1.89 (0.75, 4.74)	0.17	
**Social support**							
Strong	2 (3.5%)	55 (96.5%)		1	1		
Poor	27 (16.4%)	138 (83.6%)		5.38 (1.24, 23.39)	7.30 (1.44, 37.10)	0.01	
Moderate	5 (2.9%)	165 (97.1%)		0.83 (0.16, 4.42)	0.87 (0.14, 5.25)	0.88	

Note: Hosmer lem show test = 0.72

**Abbreviation:** AOR, Adjusted odds ratio; COR, Crude Odds Ratio; CI, Confidence Interval; COVID-19, Corona Virus Disease 2019

### Factors associated with behavioral aggression

During multiple regression analysis, male gender, knowledge about COVID-19, and substance use were significantly and positively associated with mean overt aggression score. The mean score of the overt aggression scale of male participants was increased by three units (95%CI: 1.35, 4.70) as compared to female participants. Additionally, the study participants who developed COVID-19 symptoms and had poor knowledge about COVID-19 had their overt aggression score increased by 2.5 units (95%CI: 1.26, 8.24) and 1.8 units (95%CI: 1.09, 3.41) respectively as compared with those who had no COVID-19 symptoms and had a good understanding. The average score of aggression was 1.7 (95% CI: 1.23, 6.47) higher for those who used substances (Table 6).

**Table 6:** Multiple linear regression analysis of factors associated with behavioral aggression among populations within institutional quarantine and isolation centers of COVID-19 in eastern Ethiopia, 2022.

Variables	β coefficient (95% CI)	P-values
**Sex**		
Female	0	0
Male	3.00 (1.35, 4.70)	<0.001
**Age**	-0.01 (-0.12, 0.09)	0.120
**Marital status**		
Married	0	0
Never married	-1.12 (-3.35, 1.12)	0.430
Divorced/widowed/separated	3.90 (-0.25, 8.10)	0.320
**Educational status**		
College and above	0	0
Secondary school	1.30 (-1.08, 3.55)	0.360
Primary school	0.55 (− 0.42, 1.52)	0.412
Able to write and read	1.37 (-1.20, 3.90)	0.810
Unable to write and read	− 0.71 (− 1.34, 0.49)	0.250
**Presence of COVID-19 symptoms**		
No	0	0
Yes	2.50 (0.26, 4.24)	0.14
**Knowledge about COVID-19**		
Good	0	0
Poor	1.87 (1.09, 3.41)	0.03
**Social support**		
Strong	0	0
Moderate	-0.03 (-0.02, 0.05)	0.21
Poor	2.10 (0.27, 4.35)	0.23
**Substance use**		
No	0	0
Yes	1.71 (1.23, 6.47)	0.03

NOTE: Model summary: R^2^ = 93.2%, adjusted R^2^ = 89.2%

Abbreviations: COVID-19, Corona Virus Disease 2019

## Discussion

The current study revealed the suicidal and aggressive related thoughts and behavior of suspected cases of the COVID-19 pandemic in eastern Ethiopia. The prevalence of suicidal-related thoughts and behavior was 8.7%. Of this, 21.9%, 4.8%, and 2.3% had suicidal ideation, plan, and attempt respectively. The one-year prevalence was 18.0%. These findings were higher than a meta-analysis study conducted among 308,596 participants across 54 studies that reported the pooled prevalence of suicide ideation (10.81%) and attempts (4.68%) [23]. The study further demonstrates that the mean total score of behavioral aggression was found to be 2.45±5.90 and about one-fourth (20.4%) of the participants scored above the mean of overt aggression scale.

The current study reported that women were facing a higher suicidal burden than men during the COVID-19 pandemic. This finding was in line with studies done in Bangladesh [24], Japan [25], and India [26]. Numerous potential explanations are there for this gender difference. The first one is domestic/gender violence. As shown in different countries, restrictions on movement, like quarantine, and isolation, increase the violence against women [27–29], which is identified to raise suicidal ideation and behaviors in women [30]. The findings of this study call for extra efforts to defend women from gender violence. Secondly, being without a job or a decline in job and a considerable raise in psychological distress may increase suicide rates [27]. United Nations Women has warned about the unequal increase in joblessness among women during the COVID-19 pandemic [31].

As supported by a study done in Bangladesh [12], individuals who had common mental disorders were 6 times more prone to develop suicidal-related behavior. Several psycho-pathological factors like depression, fears, stress, and insomnia will significantly intensify the risk of suicidal behavior [24, 32, 33], and about 90% of the suicide occurrences were attributed to mental disorders [34]. Therefore, to decrease suicides during the period of the COVID-19 pandemic, it is essential to reduce stress, anxiety, and aloneness.

Participants who had COVID-19 signs and symptoms were more likely to have suicidal behavior as compared to the participants who remains asymptomatic. The pandemic-associated issues might have major credit to suicidality by aggravating the mental health problems. Therefore, there is a call for critical commencement of community-based awareness programs, facilitation of genuine, consistent, and updated information, and strict monetization of propaganda, misinformation, conspiracy theories, etc., related to the pandemic [35]. These strategies can boost community understanding and protective behaviors and reduce their fear regarding COVID-19; as a result, decrease suicidality.

Having less social support was also a predictor of suicidal thoughts and behavior. This result was consistent with other studies [36–38]. The likely reason is that suspected individuals who were in centers are more likely to contract the disease and their close family and friends may be concerned about being infected, resulting in relatively less support. This may lead to suicide [39]. Therefore, close consideration should be given to enhancing the mental coping skill of those populations in quarantine and isolation to improve their psychological level.

The study further demonstrates that the mean total score of behavioral aggression was found to be 2.45±5.90 and about one-fourth (20.4%) of the participants scored above the mean of overt aggression scale. This finding shows COVID-19 stress was considerably related to physical and psychological aggression. This finding was consistent with a study done in the USA [14]. Following this, being male, having a low level of knowledge about COVID-19, and substance use were significantly and positively associated with mean overt aggression score. The relationship between substance use and aggression is complex and is indicative rather than conclusive [40, 41].

### Limitations of the study

The study has some limitations. Considering the limited availability of resources and the urgent or alarming effect of the COVID-19 pandemic outbreak, we implemented the convenience sampling technique. The present study used a cross-sectional design and relied on participant self-report.

### Conclusion

The current study revealed that suicidal behavior and aggression were prevalent among suspected cases of COVID-19. Female gender, having common mental disorders, manifesting the symptoms of COVID-19, and poor social support were significantly associated with suicide whereas male gender, knowledge about COVID-19, and substance use were significantly and positively associated with mean overt aggression score. Therefore, this study would help in developing targeted mental well-being strategies for those who are in need. Policymakers and helping professionals are advised that suicide behaviors are alarmingly common during the COVID-19 pandemic and vary based on gender, psychopathological and psychosocial factors.

## Supporting information

S1 File Data set used to determine the suicidal and aggressive behavior among populations within institutional quarantine and isolation centers of COVID-19 in eastern Ethiopia. (SAV)

## References

[1] Ahorsu DK, Lin C-Y, Imani V, Saffari M, Griffiths MD, Pakpour AHJIjomh, et al. The fear of COVID-19 scale: development and initial validation. 2020:1–9. 10.1007/s11469-020-00270-8 PMC7100496 32226353

[2] Cucinotta D, Vanelli M. WHO Declares COVID-19 a Pandemic. Acta bio-medica: Atenei Parmensis. 2020;91(1):157–60. doi: 10.23750/abm.v91i1.9397 32191675 PMC7569573

[3] Chowdhury R, Heng K, Shawon MSR, Goh G, Okonofua D, Ochoa-Rosales C, et al. Dynamic interventions to control COVID-19 pandemic: a multivariate prediction modelling study comparing 16 worldwide countries. 2020;35(5):389–99. doi: 10.1007/s10654-020-00649-w 32430840 PMC7237242

[4] Huang J, Zhang L, Liu X, Wei Y, Liu C, Lian X, et al. Global prediction system for COVID-19 pandemic. 2020;65(22):1884. doi: 10.1016/j.scib.2020.08.002 32837765 PMC7396206

[5] Cheung YT, Chau PH, Yip PS. A revisit on older adults suicides and Severe Acute Respiratory Syndrome (SARS) epidemic in Hong Kong. International journal of geriatric psychiatry. 2008;23(12):1231–8. doi: 10.1002/gps.2056 18500689

[6] Zandifar A, Badrfam RJAjop. Iranian mental health during the COVID-19 epidemic. 2020;51. doi: 10.1016/j.ajp.2020.101990 32163908 PMC7128485

[7] Xiang Y-T, Yang Y, Li W, Zhang L, Zhang Q, Cheung T, et al. Timely mental health care for the 2019 novel coronavirus outbreak is urgently needed. 2020;7(3):228–9. doi: 10.1016/S2215-0366(20)30046-8 32032543 PMC7128153

[8] Gunnell D, Appleby L, Arensman E, Hawton K, John A, Kapur N, et al. Suicide risk and prevention during the COVID-19 pandemic. 2020;7(6):468–71. doi: 10.1016/S2215-0366(20)30171-1 32330430 PMC7173821

[9] Pramukti I, Strong C, Sitthimongkol Y, Setiawan A, Pandin MGR, Yen C-F, et al. Anxiety and suicidal thoughts during the COVID-19 pandemic: cross-country comparative study among Indonesian, Taiwanese, and Thai university students. 2020;22(12):e24487. doi: 10.2196/24487 33296867 PMC7772053

[10] Stuckler D, Basu S, Suhrcke M, Coutts A, McKee MJTL. The public health effect of economic crises and alternative policy responses in Europe: an empirical analysis. 2009;374(9686):315–23. doi: 10.1016/S0140-6736(09)61124-7 19589588

[11] O’Connor RC, Kirtley OJ. The integrated motivational–volitional model of suicidal behaviour. Philosophical Transactions of the Royal Society B: Biological Sciences. 2018 Sep 5;373 doi: 10.1098/rstb.2017.0268 30012735 PMC6053985

[12] Mamun MA. Suicide and suicidal behaviors in the context of COVID-19 pandemic in Bangladesh: a systematic review. Psychology Research and Behavior Management. 2021;14:695. doi: 10.2147/PRBM.S315760 34113185 PMC8185458

[13] Lin CY, Alimoradi Z, Ehsani N, Ohayon MM, Chen SH, Griffiths MD, et al. Suicidal ideation during the CoViD-19 pandemic among a large-scale Iranian sample: the roles of generalized trust, insomnia, and fear of CoViD-19. InHealthcare. 2022; 10 (1):93. doi: 10.3390/healthcare10010093 35052258 PMC8775802

[14] Killgore WD, Cloonan SA, Taylor EC, Anlap I, Dailey NSJJoadr. Increasing aggression during the COVID-19 lockdowns. J Affect Disord Rep.2021;5:100163. doi: 10.1016/j.jadr.2021.100163 34075370 PMC8161773

[15] Lipshitz M, Ekström I. Domestic violence and its reverberations: Nova Publishers; 2006.

[16] Buzawa ES, Buzawa CGJJoPA, Management. What does research suggest are the primary risk and protective factors for intimate partner violence (IPV) and what is the role of economic factors? 2013:128–37.

[17] Youngmann R, Zilber N, Workneh F, Giel RJTP. Adapting the SRQ for Ethiopian populations: a culturally-sensitive psychiatric screening instrument. 2008;45(4):566–89. doi: 10.1177/1363461508100783 19091726

[18] Humeniuk R, Ali R, Babor TF, Farrell M, Formigoni ML, Jittiwutikarn J, et al. Validation of the alcohol, smoking and substance involvement screening test (ASSIST). 2008;103(6):1039–47. doi: 10.1111/j.1360-0443.2007.02114.x 18373724

[19] Kocalevent R-D, Berg L, Beutel ME, Hinz A, Zenger M, Härter M, et al. Social support in the general population: standardization of the Oslo social support scale (OSSS-3). 2018;6(1):1–8. doi: 10.1186/s40359-018-0249-9 30016997 PMC6050647

[20] Osman A, Bagge CL, Gutierrez PM, Konick LC, Kopper BA, Barrios FXJA. The Suicidal Behaviors Questionnaire-Revised (SBQ-R): validation with clinical and nonclinical samples. 2001;8(4):443–54. doi: 10.1177/107319110100800409 11785588

[21] Cohen IL, Tsiouris JA, Flory MJ, Kim S-Y, Freedland R, Heaney G, et al. A large scale study of the psychometric characteristics of the IBR modified overt aggression scale: Findings and evidence for increased self-destructive behaviors in adult females with autism spectrum disorder. 2010;40(5):599–609. doi: 10.1007/s10803-009-0908-z 19941156

[22] Chukwujekwu D, Stanley PJNjom. The Modified Overt Aggression Scale: how valid in this environment? 2008;17(2):153–5. doi: 10.4314/njm.v17i2.37373 18686830

[23] Dubé JP, Smith MM, Sherry SB, Hewitt PL, Stewart SHJPr. Suicide behaviors during the COVID-19 pandemic: a meta-analysis of 54 studies. 2021;301:113998. doi: 10.1016/j.psychres.2021.113998 34022657 PMC9225823

[24] Rahman ME, Al Zubayer A, Bhuiyan MRAM, Jobe MC, Khan MKAJH. Suicidal behaviors and suicide risk among Bangladeshi people during the COVID-19 pandemic: an online cross-sectional survey. 2021;7(2):e05937. doi: 10.1016/j.heliyon.2021.e05937 33615003 PMC7879153

[25] Nomura S, Kawashima T, Yoneoka D, Tanoue Y, Eguchi A, Gilmour S, et al. Trends in suicide in Japan by gender during the COVID-19 pandemic, up to September 2020. 2021;295:113622. doi: 10.1016/j.psychres.2020.113622 33290942

[26] Dsouza DD, Quadros S, Hyderabadwala ZJ, Mamun MAJPr. Aggregated COVID-19 suicide incidences in India: Fear of COVID-19 infection is the prominent causative factor. 2020;290:113145. doi: 10.1016/j.psychres.2020.113145 32544650 PMC7832713

[27] Every-Palmer S, Jenkins M, Gendall P, Hoek J, Beaglehole B, Bell C, et al. Psychological distress, anxiety, family violence, suicidality, and wellbeing in New Zealand during the COVID-19 lockdown: A cross-sectional study. 2020;15(11):e0241658. doi: 10.1371/journal.pone.0241658 33147259 PMC7641386

[28] Usher K, Bhullar N, Durkin J, Gyamfi N, Jackson DJIjomhn. Family violence and COVID‐19: Increased vulnerability and reduced options for support. 2020. doi: 10.1111/inm.12735 32314526 PMC7264607

[29] Van Gelder N, Peterman A, Potts A, O’Donnell M, Thompson K, Shah N, et al. COVID-19: Reducing the risk of infection might increase the risk of intimate partner violence. 2020;21. 10.1016/j.eclinm.2020.100348 PMC7151425 32292900

[30] Ellsberg M, Jansen HA, Heise L, Watts CH, Garcia-Moreno CJTl. Intimate partner violence and women’s physical and mental health in the WHO multi-country study on women’s health and domestic violence: an observational study. 2008;371(9619):1165–72. 10.1016/S0140-6736(08)60522-X 18395577

[31] Women U, Snyder DJG, COVID-19 Policy Brief Series. UN Women NY. COVID-19 and the care economy: Immediate action and structural transformation for a gender-responsive recovery. 2020.

[32] Mamun MA, Sakib N, Gozal D, Bhuiyan AI, Hossain S, Bodrud-Doza M, et al. The COVID-19 pandemic and serious psychological consequences in Bangladesh: a population-based nationwide study. 2021;279:462–72. doi: 10.1016/j.jad.2020.10.036 33120247 PMC7568472

[33] Tasnim R, Islam MS, Sujan MSH, Sikder MT, Potenza MNJC, review ys. Suicidal ideation among Bangladeshi university students early during the COVID-19 pandemic: Prevalence estimates and correlates. 2020;119:105703. 10.1016/j.childyouth.2020.105703 PMC7654299 33204046

[34] Klonsky ED, May AM, Saffer BYJArocp. Suicide, suicide attempts, and suicidal ideation. 2016;12:307–30. doi: 10.1146/annurev-clinpsy-021815-093204 26772209

[35] Hasan MT. Addressing the COVID-19 related stigma and discrimination: A fight against infodemic in Bangladesh. 2020.

[36] Asfaw H, Yigzaw N, Yohannis Z, Fekadu G, Alemayehu YJPo. Prevalence and associated factors of suicidal ideation and attempt among undergraduate medical students of Haramaya University, Ethiopia. A cross sectional study. 2020;15(8):e0236398. doi: 10.1371/journal.pone.0236398 32785295 PMC7423400

[37] Bitew H, Andargie G, Tadesse A, Belete A, Fekadu W, Mekonen TJDr, et al. Suicidal ideation, attempt, and determining factors among HIV/AIDS patients, Ethiopia. 2016;2016. doi: 10.1155/2016/8913160 27747101 PMC5055996

[38] Fang X-H, Wu L, Lu L-S, Kan X-H, Wang H, Xiong Y-J, et al. Mental health problems and social supports in the COVID-19 healthcare workers: a Chinese explanatory study. 2021;21(1):1–8. doi: 10.1186/s12888-020-02998-y 33435867 PMC7802988

[39] Mamun MA, Bodrud-Doza M, Griffiths MD. Hospital suicide due to non-treatment by healthcare staff fearing COVID-19 infection in Bangladesh? Asian journal of psychiatry. 2020;54:102295. doi: 10.1016/j.ajp.2020.102295 32682303 PMC7836357

[40] Fagan JJHA. Interactions among drugs, alcohol, and violence. 1993;12(4):65–79. doi: 10.1377/hlthaff.12.4.65 8125449

[41] Johnson EM, Belfer MLJJoHCftP, Underserved. Substance abuse and violence: cause and consequence. 1995;6(2):113–21. 10.1353/hpu.2010.0578 7795023

