# Supplementary material for: Suicidal and aggressive behavior among populations within institutional quarantine and isolation centers of COVID-19 in eastern Ethiopia: A cross-sectional study
Source: PLoS One. 2023 Jun 27;18(6):e0287632. doi: 10.1371/journal.pone.0287632 (PMC10298757; doi:10.1371/journal.pone.0287632)
Supplement: Attachment — Submitted filename: Rebuttal letter, second revision.docx [file pone.0287632.s003.docx]

**POINT-BY-POINT REBUTTAL LETTER**

We were pleased to have an opportunity to revise our manuscript entitled “**Suicidal and aggressive behavior among populations within institutional quarantine and isolation centers of COVID-19 in eastern Ethiopia: A cross-sectional study**” for the second time. In the revised manuscript, we have carefully considered journal requirements, the academic editor's and reviewer's suggestions and comments and we have tried to address it accordingly. The academic editor’s and reviewer’s comments were very helpful overall, and we are appreciative of such constructive feedback on our original submission. After addressing the issues raised, we feel the quality of the paper is much improved.

Sincerely,

On behalf of all authors,

Tadesse Misgana

**Journal Requirements:**

Please review your reference list to ensure that it is complete and correct. If you have cited papers that have been retracted, please include the rationale for doing so in the manuscript text, or remove these references and replace them with relevant current references. Any changes to the reference list should be mentioned in the rebuttal letter that accompanies your revised manuscript. If you need to cite a retracted article, indicate the article’s retracted status in the References list and also include a citation and full reference for the retraction notice.

***Author’s response: We have checked all the references and it is complete and correct. We didn’t use the papers that have been retracted. Thank you.***

**Editor Comments:**

1. Thoroughly edit your work as suggested in reviewers' comments

***Author’s response: We seriously took all the comments raised by the editor’s and reviewers, and we thoroughly edit the manuscript as suggested. Thank you.***

2. Provide justification for using all subscales of the Modified Overt Aggression Scale

***Author’s response: Dear editor and reviewer, thank you for your concern. Some of the suspected individuals who were in isolation centers show all the types of aggression including aggression toward the self and properties. For instance, scratches skin, pulls hair out, hits self, hits fists into walls, throws self onto floor; Inflicts minor cuts, bruises, burns, or welts on self, and inflicts major injury on self, which are the manifestation of auto aggression. Again others showed aggression towards property like slamming door, rip clothing, urinates on floor; throwing objects down, kicks furniture, defaces walls; Breaks objects, smashes windows; and Sets fires. Because of such behaviors, we decided to assess all the 4 sub scales of MOAS. Thank you.***

3. Present your tables in APA format

***Author’s response: We have accepted the comment and we presented the tables in APA format in the revised manuscript. Thank you.***

**Reviewer’s comment:**

Reviewer #4:

1. Language: Use and editing

Your work is full of words that should not be used where they are. You need to make the necessary adjustments. See below;
Line 68: replace the word "conceded"
Line 78: be clear on what you mean by "socio-economic crisis"
Line 81: What does "the other recognized precipitating risk factors of suicide could be negatively affected" mean?
Line 125 (in Methods): What is the outcome variable?
Line 136: replace the word "choose"
Line 138: What does "data were collected through face-to-face and self-reporting" mean?
Line 248-9: capital letters mid-sentence

***Author’s response: We have accepted all the comments raised regarding language use and editing, and we amended our manuscript as highlighted in the revised version. Thank you so much.***
2. Instruments
The MOAS has 4 categories. What justification exists for using ALL sub-scales of the scale especially auto and property aggression for people in isolation?

***Author’s response: Some of the suspected individuals who were in isolation centers show all the types of aggression. For instance, they scratches skin, pulls hair out, hits self, hits fists into walls, throws self onto floor; Inflicts minor cuts, bruises, burns, or welts on self, and inflicts major injury on self which are the manifestation of auto aggression. Again others showed aggression towards property like slamming door, rip clothing, urinates on floor; throwing objects down, kicks furniture, defaces walls; Breaks objects, smashes windows; and Sets fires. Because of such behaviors, we decided to assess all the 4 sub scales of MOAS. Thank you.***
3.Pilot
Comment on why a pre-test was done.

***Author’s response: We did the pre test in order to evaluate the acceptability and applicability of the procedures and tools. We have added this reason in the revised manuscript. Thank you.***

4. Analysis

a. Why was data on substance use collected given that it referred to 3 months pre-isolation? Of what benefit was/is the data to the study? What objective does the data respond to?

***Author’s response: We used a standardized WHO screening questionnaire Alcohol, Smoking, and Substance Involvement Screening Test (ASSIST) which define the current substance user as “When participants used at least one specified substance (for non medical purposes) in the last three months” and Ever substance users as “When participants used specified substance (for non-medical purposes) even once in their lifetime”. We assessed the substance use as independent variables since it known to be the risk factors for both suicidal and aggressive behavior.* *Thank you.***

b. Prepare table in APA format.

***Author’s response: We have accepted the comment and we presented the tables in APA format. Thank you.***
